# Is Digital Drying an Effective Method for Infrared Spectroscopy of Gaseous Biofluid?

**DOI:** 10.1021/acs.analchem.5c04584

**Published:** 2025-10-28

**Authors:** Kiran Sankar Maiti, Juergen Hauer, Susmita Roy

**Affiliations:** † TUM School of Natural Sciences, Department of Chemistry, 9184 Technical University of Munich, 85748 Garching, Germany; ‡ School of Medicine and Health, Center for Digital Health and Technology, Klinikum Rechts der Isar, Department of Orthopaedics and Sports Orthopaedics, 9184 Technical University of Munich, Ismaninger Str. 22, 81675 Munich, Germany

## Abstract

In recent years, gaseous biofluid analysis has gained popularity as a diagnostic approach due to the noninvasive collection of gaseous bioprobes. Among many biofluids, human exhaled breath stands out as a promising option, providing strong evidence of volatile biomarkers for various diseases. However, identifying these biomarkers is challenging due to the high concentration of water vapor in exhaled breath. Various experimental techniques, such as mass spectrometry, electronic nose, infrared spectroscopy, etc., have demonstrated their potential as diagnostic tools. However, water vapor in exhaled breath poses a significant obstacle to metabolite identification across nearly all experimental methods, particularly in infrared spectroscopy-based diagnostics. Nevertheless, in addition to physical removal, careful analysis of the spectral behavior of water molecules enables highly accurate identification of disease-specific biomarkers using infrared spectroscopy. This study presents a comprehensive qualitative analysis of the infrared spectral features of gaseous water molecules.

## Graphical abstract

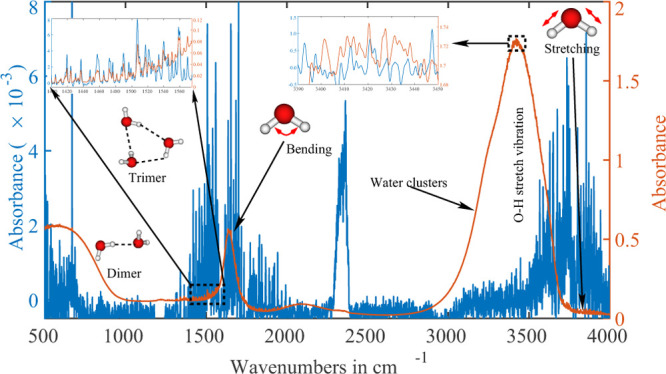

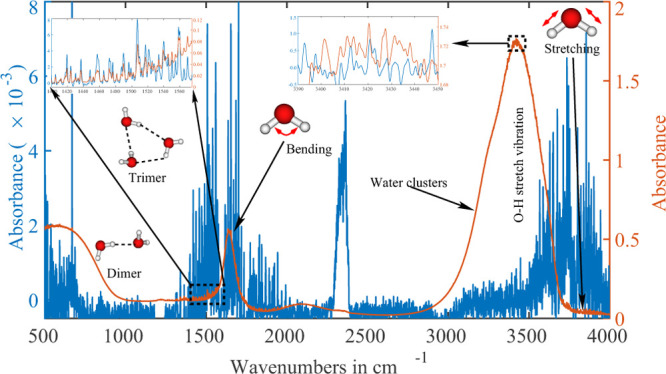

## Introduction

Water is an essential substance for biological processes, serving as both a medium and a reagent.^1,2^ In humans, nearly 60% of body weight is composed of water.^3^ As a result, most biological samples collected for diagnostic purposes contain significant amounts of water.^4–6^ While this abundance of water can benefit many diagnostic techniques,^7,8^ it presents a significant challenge, particularly in metabolic analysis using spectroscopy.^9,10^ The high electron affinity of the oxygen atom in water molecules makes them highly reactive, enabling hydrogen bonding with biomolecules.^11–13^ Consequently, in spectroscopic analysis, the spectral data of the target molecule are influenced by the cumulative effect of water. Additionally, water spectra generate a strong background that can obscure molecular spectral signals. For a successful metabolic analysis, it is essential to remove the spectroscopic effects of water from the acquired data of biological samples. Therefore, understanding the properties and behavior of water is essential for metabolite-based diagnostics.

“Water—an enduring enigma,” as science writer Philip Ball aptly remarked in a Nature article: “No one really understands water”.^14,15^ In fact, the deeper our understanding grows, the more puzzling it becomes. The extraordinary hydrogen-bonding capability of water molecules enables the formation of dynamic water clusters of varying sizes.^16–18^ These clusters are in a constant state of flux, undergoing continuous hydrogen bond formation and breaking processes.^19,20^ This dynamic nature of water clusters results in highly complex molecular spectral characteristics in both liquid and gas phases.^21^ Moreover, water molecules have an exceptionally small moment of inertia during rotation, resulting in intricate vibrational–rotational spectra in the vapor phase, with tens of hundreds to thousands of absorption lines.^22,23^ These features present significant challenges for metabolite-based diagnostics, in particular, for gas phase biofluids.

Gas-phase biofluid analysis is gaining popularity as a diagnostic method, utilizing biofluids such as exhaled breath, and the headspace of urine, blood, and saliva.^24–27^ These gaseous biofluids typically contain water vapor at or near their saturation point at room temperature, posing challenges for most existing experimental methods.^9,10^ The issues are particularly pronounced in infrared spectroscopy-based diagnostics. Water is well-known for its strong absorption of infrared light across a broad spectral region. Consequently, water absorption lines often overlap with molecular absorption bands, thereby altering the spectral features of the molecules of interest. In the gas phase, both metabolites and water produce narrow absorption bands, making the separation of overlapping spectra particularly challenging.^28–30^ Furthermore, metabolites in gaseous biofluids are typically present at trace concentrations, resulting in extremely weak absorption peaks. In contrast, water, which presents at its saturated vapor pressure, generates significantly stronger absorption spectra. This disparity often causes molecular spectra to be obscured by water spectra, rendering metabolic analysis nearly impossible.

Nevertheless, careful sample preparation and advanced data processing^31,32^ can mitigate many of these challenges, enabling infrared spectroscopy to serve as a viable diagnostic tool for gaseous biofluids. For instance, water vapor can be physically removed significantly from gaseous biofluids without compromising the metabolic content.^33^ This not only overcomes the overshading effect^9^ of water across a broad spectral range but also significantly reduces the number of water spectral lines. This approach has been successfully employed to identify and quantify metabolites in human exhaled breath, urine headspace, and bacterial headspace analysis.^34,35^

Further improvements can be achieved during the data analysis. While focusing on water absorption-free spectral windows is an ideal approach for metabolite analysis,^36^ the extremely broad absorption bands of water often result in the loss of valuable metabolic information. Consequently, this limitation hinders comprehensive multimetabolic analysis, which is an essential component for enhancing diagnostic accuracy. The concept of “digital drying,” or computational removal of water absorption bands, is a significant area of discussion in infrared spectroscopy.^37,38^ Although ideal in theory, straightforward digital subtraction is ineffective due to the ever-changing spectral behavior of water.^11,15^ However, a deeper understanding of water spectra enables the extraction of meaningful metabolite information, thereby enhancing the diagnostic accuracy. In this article, we discuss the key issues associated with water vapor interference and propose practical solutions to mitigate its impact on infrared spectroscopic analysis of gaseous biofluids.

## Experimental Procedure

To gain deeper insight into the unique behavior of water molecules in the gas phase, varying concentrations of water vapor samples were prepared, and their infrared absorption spectra were measured within the spectral range of 500–4000 cm^–1^. To ensure consistency, all samples were analyzed at room temperature and a uniform pressure of 500 mbar. Nitrogen gas with 99.99% purity was added into the water vapor to maintain constant pressure within the sample cell.

Figure 1 presents a schematic representation of the experimental setup. Except for the measurement unit, all sample handling components shown in the figure are custom-built. The sample collection unit is designed to collect water vapor and, if necessary, mix different gases in desired proportions.

**Figure 1:**
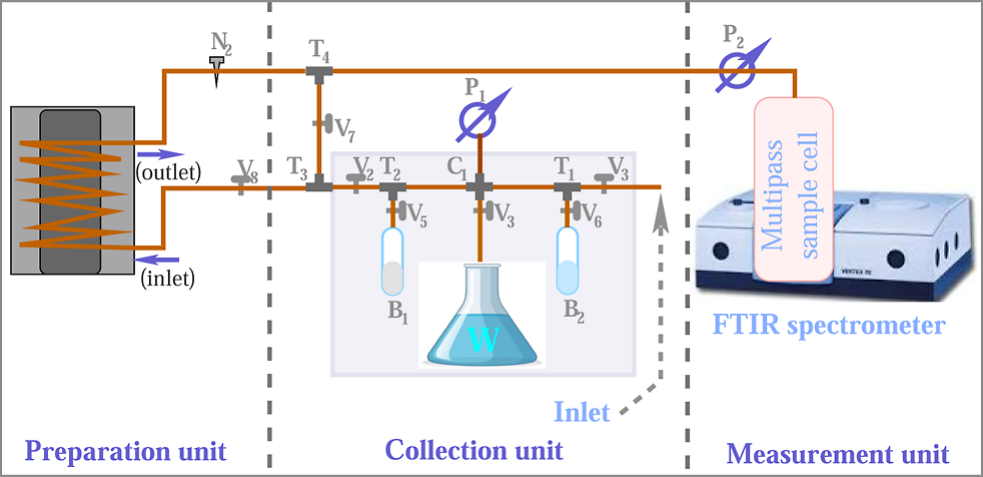
Experimental setup to prepare precise concentration of water vapor. The setup is equipped with two vacuum pumps to clean the sample path and cell.

The sample collector consists of a 50 cm copper tube with an inner diameter of 3 mm and an outer diameter of 6 mm, closed at both ends with two valves (**V**_**1**_ and **V**_**2**_) that control the inlet and outlet. A cross-connector (C_1_) is positioned at the center of the tube, with its two open ends closed by valves (**V**_**3**_ and **V**_**4**_) and fitted with ISO KF-16 adapters to facilitate both evacuation and water vapor collection. A turbo pump (P_1_) is employed to evacuate the sample collection and preparation units to a pressure of 10^–5^ mbar. Additionally, two T-connectors (T_1_ and T_2_) are attached to the copper tube between the cross-connector (C_1_) and the valves (**V**_**1**_ and **V**_**2**_). Each T-connector has a third arm fitted with a valve (**V**_**5**_ or **V**_**6**_) and an ISO KF-16 adaptor, allowing for the attachment of sample bottles via a flange. Two different sample bottles (B_1_ and B_2_) are connected to mix the sample gases independently.

Another T-connector (T_3_) is installed at the outlet of the copper tube. One arm of this connector is linked directly to the sample cell via a control valve (**V**_**7**_), while the other arm connects to the water condenser through another control valve (**V**_**8**_). These control valves enable the sample to be directed either into the cell or through the condenser for water vapor suppression. All valves, T-connectors, and cross-connectors used in the setup are sourced from Swagelok.

The water condenser is allowed to prepare water vapor samples with specific concentrations. It is a 12 m long copper tube with the same inner and outer diameters as used for the sample collector, placed inside a closed cylindrical metal chamber (diameter: 20 cm and height: 20 cm) in the form of a spiral. Two ends of the copper spiral are fed through the chamber wall. One end (inlet) of the spiral is connected with the sample collector and the other end (outlet) with the sample cell using the same kind of copper tube. The chamber is filled with silicon-based bath fluid with an operating temperature of −95° to +55 °C. The temperature of the chamber is precisely controlled by an ultralow refrigerated circulator (FW95-SL, Julabo Labortechnik GmbH).

A Fourier transform infrared (FTIR) spectrometer (Bruker Vertex 70, Bruker Optics GmbH, Germany) was used to collect the infrared absorption spectra of water vapor. The spectrometer was equipped with a White cell featuring a 4 m optical path length and a 2 L volume (Bruker Optics GmbH, Germany) for containing gaseous samples during analysis. To eliminate interference from environmental water vapor, the spectrometer was purged with dry nitrogen gas, creating an overpressure inside both the spectrometer and the sample chamber. This process gradually reduced the presence of water molecules over time. Near-equilibrium conditions were achieved by maintaining a nitrogen flow rate of 200 ml/sec for 3 hours before beginning measurements. To further minimize discrepancies in the water vapor concentration between background and sample scans, a background scan was conducted immediately before each sample scan. The absorption spectra were recorded using a liquid nitrogen-cooled mercury cadmium telluride detector, ensuring a spectral resolution of 0.16 cm^–1^ for all measurements. Each acquired spectrum is averaged over 100 measurements to reduce the random instrumental and electronic noise.

## Results and Discussions

Infrared spectra of the water vapor prepared at two different temperatures are presented in Figure 2. In the first case, after evacuating both the sample collector and measurement cell, water vapor was allowed to flow directly from the water flask **W** into the sample cell by opening valve **V**_**3**_, **V**_**2**_, and **V**_**7**_. Additionally, dry nitrogen gas was introduced by opening valve **V**_**1**_ until the pressure in the sample cell reached 500 mbar. The acquired infrared spectrum is plotted in red, with the corresponding absorption strength displayed on the right side of the plot.

**Figure 2:**
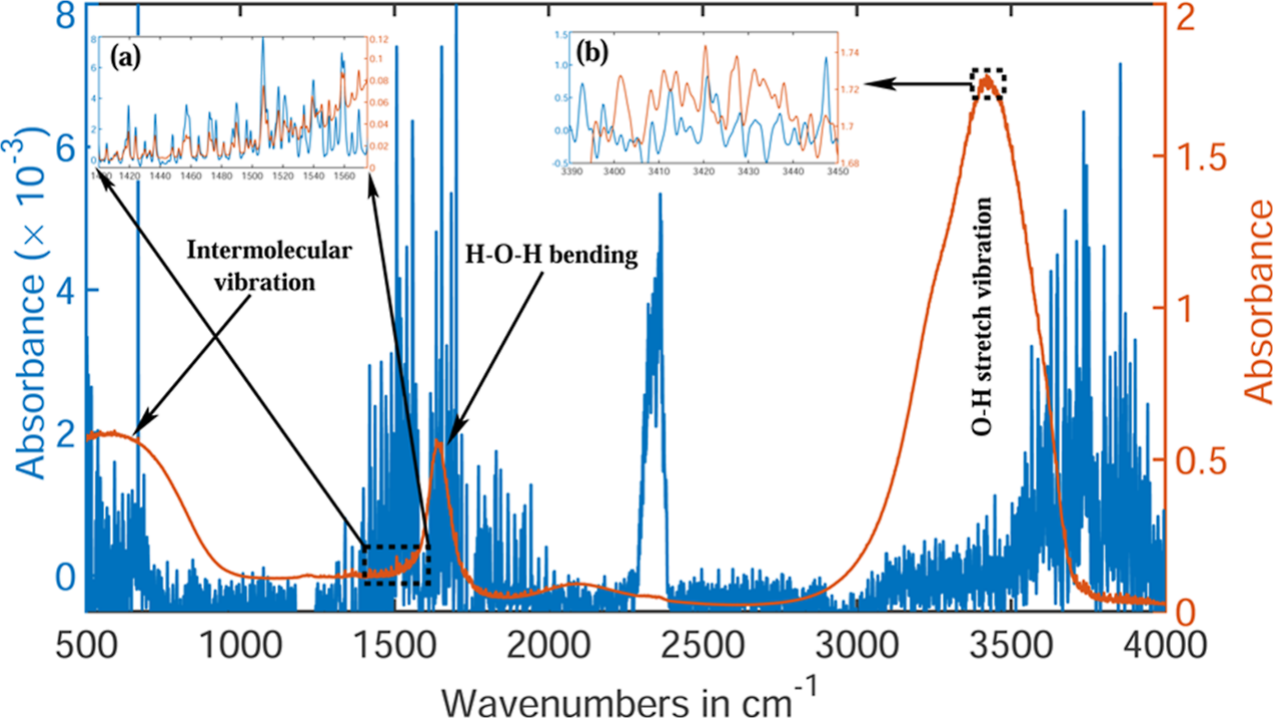
Infrared absorption spectra of water are shown at two different water vapor pressures. The continuous red line represents the spectrum at saturated vapor pressure (30 mbar), while the blue lines indicate the line spectra observed at low vapor pressure (1.035 mbar). Both spectra represent an average of 100 measurements, acquired with a spectral resolution of 0.16 cm^–1^. Two different scales are used for plotting these spectra, corresponding to their respective colors. (a) Magnified view of the H–O–H bending vibrational region of the water molecules. (b) The O–H stretching vibrational regions of water molecules are enlarged and presented. The axis labels of both the insets are identical to those of the main figure.

In the second case, water vapor was first confined within the evacuated sample collector, specifically in the volume between valves **V**_**1**_ and **V**_**2**_. It was then passed through a cold chamber (**V**_**7**_ closed and **V**_**8**_ opened) maintained at −20 °C at a controlled flow rate of 3 ml/sec. The precise control of flow was regulated using needle valve **N**_**2**_. The regulated flow ensured that the water vapor remained within the cold spiral long enough to freeze. However, some water molecules remained in the gas phase that entered the sample cell. The quantity of gaseous water molecules at a given temperature can be determined using the Clausius–Clapeyron relation.^39^ To maintain consistency with the first experiment, dry nitrogen was added to the sample until the pressure in the cell reached to 500 mbar. The infrared absorption spectrum for this case is plotted in blue, with the corresponding scale shown on the left side of the plot.

The infrared spectra of water differ significantly between these two scenarios. In the case of saturated vapor pressure (red spectrum in Figure 2), the absorption spectrum is broad and strong, whereas in the case of suppressed water vapor (blue spectrum), it consists of considerably narrower spectral lines with an intensity 3 orders of magnitude lower than for higher water concentration. This significant difference in the intensity is expected. In the first case, the sample cell was saturated with water vapor at a vapor pressure of 30 mbar (at room temperature), resulting in a high concentration of water molecules that strongly absorbed infrared light. In contrast, in the second case, a majority of the water molecules were frozen, leaving only a small fraction of the water molecules in the gas phase. At −20 °C, the vapor pressure was reduced to just 1.035 mbar, meaning far fewer water molecules reached the sample cell, leading to a significant drop in absorption strength.^33,40^

The most intense water absorption peak, centered at 3420 cm^–1^, is notably broad, with a full width at half-maximum (fwhm) of approximately 400 cm^–1^. This peak corresponds to O–H stretching vibrations,^41^ which are typically expected around 3700 cm^–1^. However, due to the high electron affinity of oxygen in water molecules, hydrogen bonding is highly probable when water molecules come into close proximity. In general, hydrogen bonding restricts vibrational motion, leading to a red shift in the vibrational frequency.^42^ The observed broad, apparent structure-free peak is a characteristic feature of liquid water.^23^ This can be explained by the following elucidation. Since the experiment involved saturated water vapor, a substantial number of water molecules came in close proximity and formed clusters of varying size through hydrogen bonding. A single water molecule can form up to four hydrogen bonds, resulting in clusters of five water molecules.^43,44^ Each of these water molecules further forms four additional hydrogen bonds with neighboring molecules, potentially leading to the formation of a fullerene-like structure.^16,45^ If the clusters are sufficiently large, they exhibit bulk liquid behavior and form water droplets.^46,47^ This clustering effect produces infrared absorption features similar to those of liquid water.

Additionally, the sample contained single water molecules, dimers, and smaller clusters,^16^ which contributed to minor absorption features. These appeared as minute features at the high-energy shoulder of the OH-peak, around 3750 cm^–1^, and at the crest of the peak^48^ (see inset (b) of Figure 2). Another distinct peak is observed in the saturated water sample around 1650 cm^–1^. It is less intense than the O–H stretch vibrational peak and significantly narrower, with a fwhm of approximately 80 cm^–1^. It originates from the H–O–H bending motion of water molecules. While initially expected near 1590 cm^–1^, hydrogen bonding induces a blue shift.^49–51^ Notably, unlike stretching vibrations, hydrogen bonding shifts the bending frequency to a higher value. The continuous and relatively broad peak further supports the presence of water droplets, as previously discussed.

A magnified view of a narrow spectral range at the left shoulder of this peak is shown as an inset (a) in Figure 2. The overlapping oscillations of the red and blue curves indicate the presence of single water molecules and small molecular clusters, in addition to water droplets in the first experimental case.

In the experiment with a decreased water vapor pressure, fewer water molecules are present in the sample, reducing the likelihood of two or more molecules coming close together. Even when water molecules interact and form hydrogen bonds, only small clusters can form. These clusters are highly fragile due to the low vapor pressure of water, causing hydrogen bonds to break easily.^12,29^ The continuous formation and breaking of hydrogen bonds result in a complex absorption spectrum in the infrared region.^19,52^ Additionally, the extremely low moment of inertia of water molecules during rotation leads to hundreds of thousands of absorption lines from freely moving water molecules. Consequently, a large number of absorption lines (blue lines) are observed in Figure 2. These absorption lines are significantly narrower than those in the previous experiment, with fwhm values ranging from fractions of a wavenumber to just a few wavenumbers. Another notable characteristic of these water lines is that many do not remain at fixed spectral positions due to collisions with other molecules. These collisions modify the ro-vibrational wave function of water molecules, causing shifts in the spectral positions of absorption lines.^53,54^ As collisions do not alter the molecular moment of inertia, the line shape remains unchanged.

The spectral shift and broadening caused by hydrogen bonding and molecular collisions can be understood by closely examining the spectra. For example, the fundamental O–H stretch vibrational peak^55^ of the water monomer is expected to be found at 3703 cm^–1^. The presence of the water monomer in both experiments is confirmed by the observed peak at 3703 cm^–1^ in both the blue and red plots in Figure 3. Both peaks exhibit equal broadening (1.5 cm^–1^), indicating that they originate from the same species—the water monomer. However, the absorption lines at around 3712 cm^–1^ differ between the blue and red plots. The red absorption line is shifted 1 cm^–1^ to lower energies and is broadened by an additional 0.5 cm^–1^ compared to the blue absorption line. This behavior is characteristic of hydrogen bonding.^56^ Therefore, it is concluded that the red absorption line originates from a hydrogen-bonded species.

**Figure 3:**
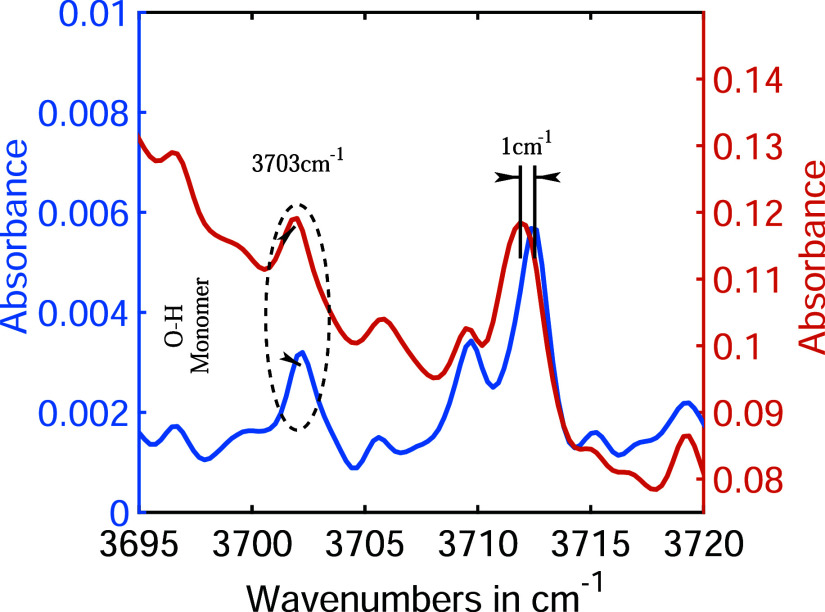
In both saturated and suppressed water vapor conditions, water monomers exhibit a fundamental O–H stretching vibrational absorption at 3703 cm^–1^. At higher vapor pressure, the O–H stretch vibration at around 3712 cm^–1^ shifts and broadens due to hydrogen bonding with other water molecules.

Another spectral region at higher frequency is magnified and is shown in Figure 4. While many absorption lines from both experiments overlap well, several exhibit varying degrees of shift and broadening. Figure 4b highlights three such peaks, each representing a different type of water clusters.

**Figure 4:**
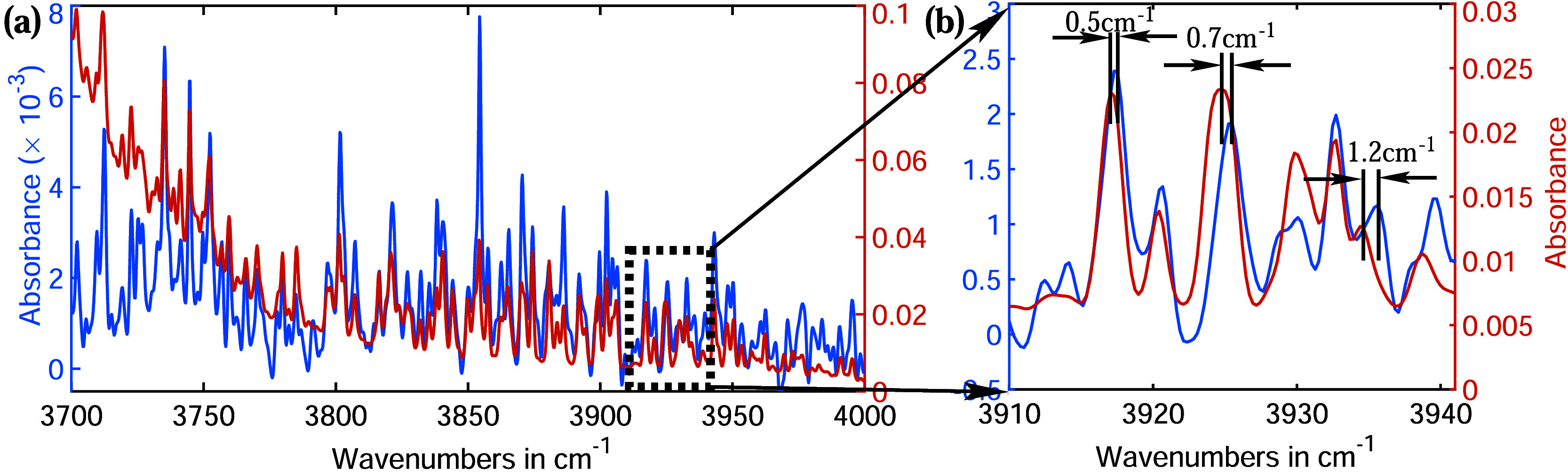
(a) The O–H stretch vibrational region of a gaseous water molecule. (b) A magnified view of the spectral region highlighting the spectral shifts caused by hydrogen bonding. These shifts vary depending on the size of the water clusters.

For instance, the absorption lines around 3917 cm^–1^ have nearly identical widths but are shifted by 0.5 cm^–1^, likely due to collisions involving water monomers, which become more probable at high vapor pressure, causing the spectral shift.^53^ In contrast, the absorption lines around 3925 cm^–1^ are shifted by 0.7 cm^–1^, with the red line also exhibiting an additional broadening of 0.5 cm^–1^. This suggests that the red line originates from a water dimer, while the blue line corresponds to a monomer.^55,57^

The absorption lines around 3935 cm^–1^ show a more significant shift of 1.2 cm^–1^, along with a notable difference in the absorption strength. This strongly indicates that the red absorption line originates from larger than water dimer. Since small water clusters are less common, their absorption strength is lower than that of the blue absorption line.^20,58^ On the contrary, the absorption lines at 3933 cm^–1^ from both experiments match perfectly in both width and spectral position. This suggests that both peaks originate from the same type of species. Until now, discussions have focused on the infrared absorption of water at relatively high vapor pressures. A key question is how the absorption spectra of water would behave if the experiment were conducted at a consistently low water vapor pressure. To investigate this, two samples were prepared by passing water vapor through a cold chamber maintained at −60 °C. At this temperature, the vapor pressure of water drops to 0.01 mbar, which is 2 orders of magnitude lower than its vapor pressure at −20 °C. This substantial reduction in vapor pressure significantly decreases the absorption strength of water.

A representative spectral window is shown in Figure 5a, where the blue and red lines correspond to two separate measurements taken under identical experimental conditions. The gray line represents random noise, primarily originating from the instrument. Since the water absorption strengths are significantly higher than the noise level, the absorption lines of water remain unaffected by it. As both samples were prepared at the same temperature, an approximately equal number of water molecules were expected in the measurement chamber. Consequently, both experiments yielded similar water absorption strengths, as observed in Figure 5a. However, the spectral positions and shapes of the absorption lines varied significantly between the two experiments. For instance, the blue absorption line at around 1128 cm^–1^ is shifted by 0.25 cm^–1^ to lower energies with respect to the red absorption line, with both lines exhibiting similar spectral widths. In contrast, the next blue peak at approximately 1134 cm^–1^ is shifted 1.7 cm^–1^ to higher energies with respect to its counterpart in the red absorption line. Notably, the peak at 1136 cm^–1^ is absent in the blue spectrum, while a pronounced absorption feature appears in the red spectrum.

**Figure 5:**
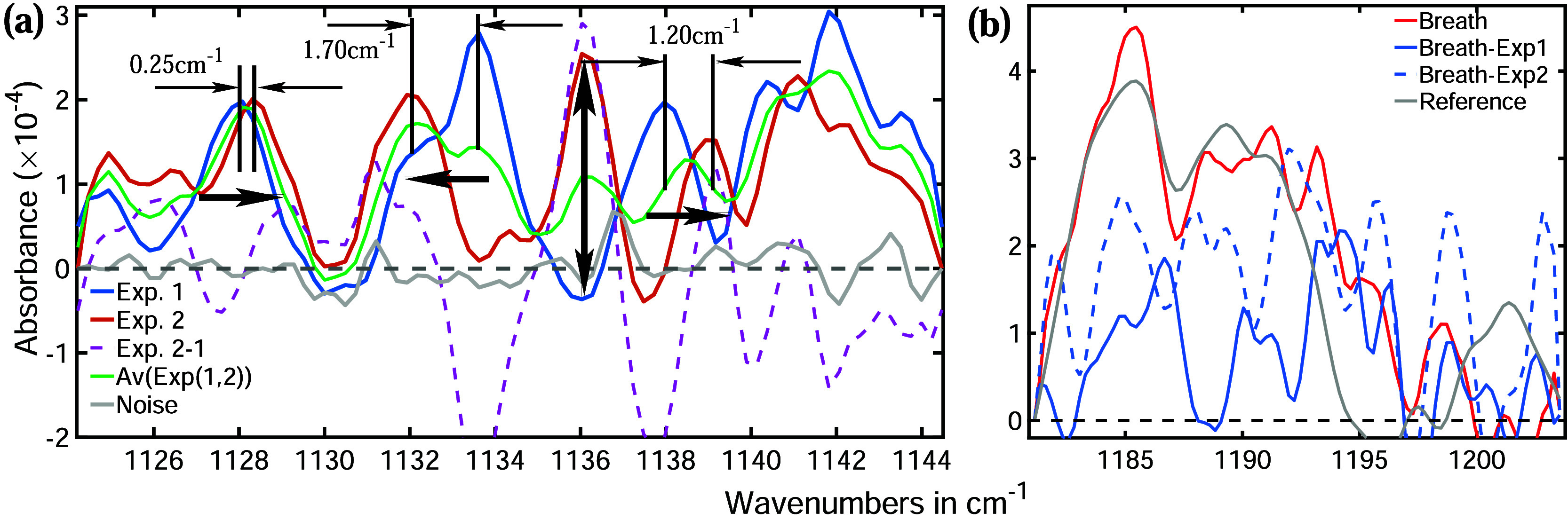
(a) The infrared absorption spectra of gaseous water were recorded at an extremely low vapor pressure of 0.01 mbar. Both the red and blue spectra were obtained under the same vapor pressure conditions. Both spectra represent an average of 100 measurements, acquired with a spectral resolution of 0.16 cm^–1^. The dotted violet line represents the difference between the two spectra, while the green line indicates their average. The absorption lines shift between experiments due to molecular collisions, which alter the ro-vibrational wave functions of water molecules. The gray line represents random instrumental noise. (b) Measured breath spectrum (red trace) in comparison with the corresponding digitally water-subtracted spectrum of the same sample (blue solid and dotted traces).

These findings indicate that the absorption lines experience unforeseeable shifts between consecutive measurements even if all experimental conditions remain the same. The observed spectral shifts are most likely caused by molecular collisions. Since molecular motion is random, individual molecules travel different distances and possess varying velocities before colliding. As a result, the impact of each collision differs, leading to varying modifications in ro-vibrational wave functions and producing variable spectral shifts across different absorption lines.

Given the random nature of the absorptive features, averaging over a larger number of spectral scans should improve the diagnostic accuracy. To evaluate this, both experimental spectra—already averaged over 100 scans—were further averaged and plotted as a green line in Figure 5a. For a few peaks, the absorption intensity decreased slightly; however, in many cases, the superposition of absorption bands led to an increase in the spectral bandwidth, further complicating the analysis of gaseous metabolites. Additionally, increasing the number of spectral scans requires more acquisition time, which slows the diagnostic process. Therefore, a trade-off must be made between diagnostic accuracy and measurement time.

This study aims to enhance the understanding of the infrared spectral behavior of water in the gas phase, enabling more accurate analysis of metabolites in gaseous biofluids and potentially improving diagnostic precision. Most metabolites in gaseous biofluids are present only in trace amounts, resulting in weak absorption spectra that are comparable to or even weaker than the absorption strength of water at a vapor pressure of 0.01 mbar. Additionally, the absorption line widths of many metabolites are similar to those of water. These suboptimal characteristics of both water and volatile metabolites complicate the analysis of gaseous biofluids. Understanding the infrared spectral characteristics of water enables the efficient separation of metabolite spectra, which is essential for developing accurate diagnostic methods. Optimal diagnostic performance was achieved using infrared spectra of biofluids devoid of water absorption lines. A widespread strategy to achieve this is the digital subtraction of the water spectrum.^59,60^ While this method is effective when molecular concentrations in the gas mixture are significantly above trace levels, unfortunately, it becomes problematic for real gaseous biofluids as exemplified by the data in Figure 5a. Due to the unpredictable spectral positions of water absorption lines, digital subtraction often creates misleading spectral profiles.

To test the feasibility of digitally subtracting the water spectrum, the spectrum from Experiment 1 was subtracted from that of Experiment 2 and plotted using a dotted violet line in Figure 5a. However, the subtraction does not produce a flat baseline; instead, several spectral features emerge prominently. For example, there is a prominent positive peak at 1136 cm^–1^, which is difficult to distinguish from water absorption lines. Additionally, two strong negative peaks are observed at 1133.5 cm^–1^ and 1137.5 cm^–1^. These negative features complicate the analysis as negative absorption is physically meaningless. Particularly in the context of statistical analyses, which are primarily undertaken for the development of diagnostic methods, artifacts may emerge and consequently reduce diagnostic accuracy. To demonstrate water-spectrum subtraction in real gaseous biofluids, a representative breath spectrum is shown in Figure 5b (red trace). A distinct double-peak feature is resolved, with absorption peaks at 1185 cm^–1^ and 1190 cm^–1^, characteristic of propyl propionate and in reasonable agreement with the reference spectrum. Two experimental water spectra were subtracted from the same breath spectrum, yielding the blue dotted and solid traces. The resulting spectra differ substantially from each other, and the characteristic double-peak feature of the original breath spectrum is largely obscured. Therefore, a straightforward digital subtraction of the water spectrum is ineffective for identifying additional metabolites with absorption strengths and spectral widths similar to those of water. However, understanding the spectral characteristics of water can aid in the effective identification of metabolite spectral features.

It is important to note that while digital subtraction of water spectra does not work straightforwardly—particularly for extreme trace gases—it can be successfully applied to liquid samples as well as to gases present at higher concentrations. In the liquid phase, water produces very broad and strong continuous absorption peaks, similar to the red curve in Figure 2. The spectral shifts caused by hydrogen bonding and molecular collisions are negligible compared to the dominant spectral bands of liquid water. Moreover, metabolites in the condensed phase typically exhibit significantly broader and stronger absorption peaks. In such cases, digital subtraction of water spectra can effectively reveal the absorption features of metabolites. For gas mixtures containing components above trace concentrations, stronger absorption bands are produced. Here, digital subtraction of water spectra is also effective, particularly in spectral windows where water absorption is relatively weak. However, identifying metabolites in gaseous biofluids requires a more refined spectral analysis strategy.

A more effective solution is the physical removal of water molecules by freezing them at extremely low temperatures. Studies have already identified more than 40 metabolites^61^ by freezing water molecules at −60 °C. For example, a representative spectral feature of a breath metabolite (black line) is shown in Figure 6. This feature corresponds to the acetone molecule. A reference spectrum of acetone (cyan line) closely matches the breath spectrum with only a few minor deviations. The water spectra within the spectral range of acetone exhibit similar features to those shown in Figure 5a. By comparing the amplitude of these small oscillations is compared with the water absorption features, a near-perfect match is observed. Given that the absorption strength of acetone is approximately six times greater than that of water, acetone can be confidently identified. The estimated concentration of acetone is 1.1 ppm (ppm). In this water-based environment, the limit of detection (LOD) is about 350 part-per-billion (ppb), which is lower than the typical level of around 500 ppb found in healthy individuals. However, most breath metabolites exist at much lower concentrations, typically in the lower ppb down to part-per-trillion (ppt) range.^62^ Their molecular absorption strengths are often comparable to—or even weaker than—the water absorption features discussed above. Lowering the sample preparation temperature reduces water vapor pressure, thereby minimizing excess water molecules, which can improve the LOD and limit the quantitation for VOCs and potentially enhance diagnostic accuracy. Given the extremely low concentration of metabolites in human breath, a further reduction in the preparation temperature by 40–50 °C is a reasonable and safe recommendation.^33^

**Figure 6:**
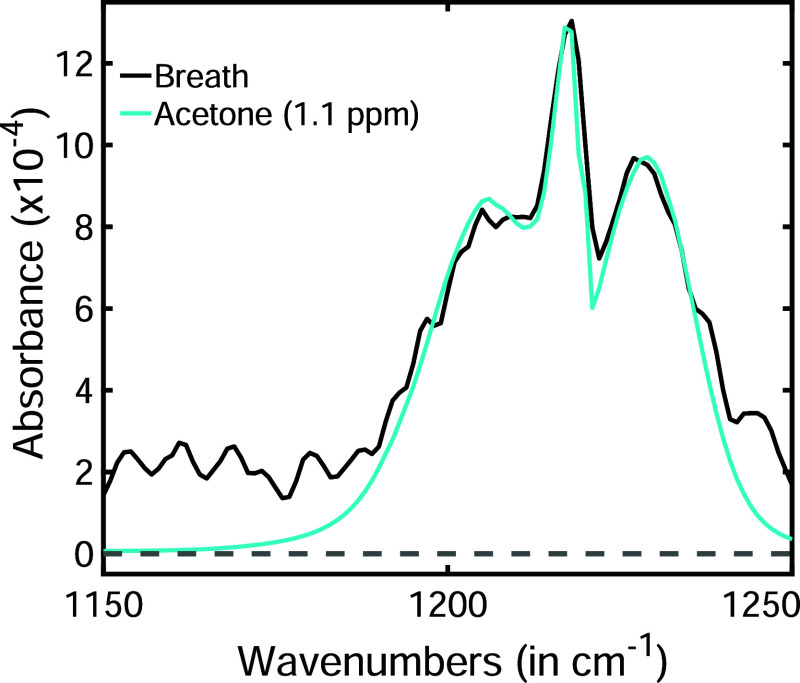
The black line represents the spectral signature of acetone in a real breath sample where vapor pressure of water is ∼0.01 mbar. The cyan line shows the reference acetone spectrum from the PNNL database. The plot has been reproduced from a previous publication with permission.^33^

An alternative approach to analyzing breath spectra is to restrict the search to water-free spectral windows. For instance, no water absorption occurs in the regions 800–1100 cm^–1^ and 2700–3300 cm^–1^. Metabolite detection within these windows can already achieve an LOD on the order of a few tens of ppb^24^. For example, 83 ppb of acetic anhydride has been reported, corresponding to an absorption band near 1005 cm^–1^. However, limiting the analysis to such windows reduces the range of detectable metabolites, which in turn affects diagnostic sensitivity, since highly accurate diagnosis relies on multimetabolite biomarkers.

Recently developed high-power lasers,^63^ quantum cascade lasers,^64^ and quartz-enhanced photoacoustic spectroscopy^65,66^ have greatly improved detection sensitivity, reaching the ppt range for breath metabolites. Nevertheless, these methods operate within very narrow spectral bands. They hold strong potential for diagnostic tool development once disease-specific biomarkers are established, but their utility for broader metabolic searches remains limited.

## Conclusions

The review explores the infrared absorption properties of water at different vapor pressures in the context of developing gaseous biofluid-based diagnostics. Although water may appear to be a simple molecule, its high electron affinity, due to the oxygen atom, gives it remarkable properties in both the liquid and gas phases. A single water molecule can form up to four hydrogen bonds with other water molecules, leading to the formation of water clusters.

In the gas phase, depending on vapor pressure, hydrogen bonding can result in structures ranging from water dimers to fullerene-like clusters, which exhibit characteristics similar to those of liquid water in infrared spectroscopy. These hydrogen bonds not only shift the spectral positions of absorption lines but also modify their shapes and broadening. At a low vapor pressure, the greater mean distance between water molecules reduces the likelihood of hydrogen bonding. However, molecular collisions shift the spectral positions of the absorption lines. Due to the random nature of molecular motion and collisions, these spectral shifts are unpredictable, meaning that water absorption lines may not appear in the same spectral position across different experiments. To average out these effects would increase the data acquisition time beyond what is feasible in a clinical context. Consequently, digitally subtracting water spectra from the infrared spectra of gaseous biofluids can produce misleading diagnostic results. Careful consideration of the spectral characteristics of water may enhance the diagnostic accuracy. In contrast, physically removing water vapor through deep cooling will significantly enhance the accuracy of infrared spectroscopic diagnostics of gaseous biofluids. Although digital drying can produce misleading results for trace metabolites in gaseous biofluids, it has proven to be effective for metabolite analysis in the liquid phase, such as blood serum analysis. It also enhances detection sensitivity for industrial and environmental gases, where concentrations are significantly higher compared with VOCs in biofluids.
